# Mobile Phone App Use Among Pregnant Women in China and Associations Between App Use and Perinatal Outcomes: Retrospective Study

**DOI:** 10.2196/29644

**Published:** 2022-01-25

**Authors:** Puhong Zhang, Huan Chen, Jie Shang, Jun Ge, Huichen Zhang, Mingjun Xu, Cui Bian, Yang Zhao, Minyuan Chen, Jane Elizabeth Hirst

**Affiliations:** 1 The George Institute for Global Health at Peking University Health Science Center Beijing China; 2 Faculty of Medicine, University of New South Wale Sydney Australia; 3 Tianjin University of Traditional Chinese Medicine Tianjin China; 4 Shijiazhuang Obstetrics and Gynaecology Hospital Shijiazhuang China; 5 Shijiazhuang Luquan People's Hospital Shijiazhuang China; 6 Department of Anesthesiology, Beijing Obstetrics and Gynecology Hospital, Capital Medical University Beijing China; 7 Gaoyang Maternal and Child Health Hospital Baoding China; 8 Nuffield Department of Women’s & Reproductive Health, University of Oxford Oxford United Kingdom; 9 The George Institute for Global Health, Central Working - Fourth Floor Translation and Innovation Hub Imperial College London London United Kingdom

**Keywords:** maternal and child health, mHealth, mobile apps, retrospective study, pregnancy outcomes

## Abstract

**Background:**

Maternal and child health (MCH)–related mobile apps are becoming increasingly popular among pregnant women; however, few apps have demonstrated that they lead to improvements in pregnancy outcomes.

**Objective:**

This study aims to investigate the use of MCH apps among pregnant women in China and explore associations with pregnancy outcomes.

**Methods:**

A retrospective study was conducted at 6 MCH hospitals in northern China. Women who delivered a singleton baby at >28 weeks’ gestation at the study hospitals were sequentially recruited from postnatal wards from October 2017 to January 2018. Information was collected on the women’s self-reported MCH app use during their pregnancy, along with clinical outcomes. Women were categorized as nonusers of MCH apps and users (further divided into intermittent users and continuous users). The primary outcome was a composite adverse pregnancy outcome (CAPO) comprising preterm birth, birth weight <2500 g, birth defects, stillbirth, and neonatal asphyxia. The association between app use and CAPO was explored using multivariable logistic analysis.

**Results:**

The 1850 participants reported using 127 different MCH apps during pregnancy. App use frequency was reported as never, 24.7% (457/1850); intermittent, 47.4% (876/1850); and continuous, 27.9% (517/1850). Among app users, the most common reasons for app use were health education (1393/1393, 100%), self-monitoring (755/1393, 54.2%), and antenatal appointment reminders (602/1393, 43.2%). Nonusers were older, with fewer years of education, lower incomes, and higher parity (*P*<.01). No association was found between *any app* use and CAPO (6.8% in nonusers compared with 6.3% in any app users; odds ratio 0.77, 95% CI 0.48-1.25).

**Conclusions:**

Women in China access a large number of different MCH apps, with social disparities in access and frequency of use. *Any app* use was not found to be associated with improved pregnancy outcomes, highlighting the need for rigorous development and testing of apps before recommendation for use in clinical settings.

## Introduction

### Background

In the past 2 decades, while maternal and child health (MCH) has greatly improved in China [1], prevention of preterm birth, neonatal asphyxia, birth defects, and low birth weight remain challenging. With mobile phone penetration growing rapidly, mobile health (mHealth) technology is being increasingly used and recognized as a tool that can improve access to, and use of, health services, including in MCH [2-6]. Thousands of MCH mobile apps are available, with millions of downloads [6-9]. Although most research in this area has focused on feasibility and acceptability studies, [9-14] mHealth could be a valuable tool for strengthening health systems [15,16].

There is evidence to support that specific apps can improve antenatal and postnatal service use [6,17-19], diet and gestational weight gain [20-22], blood glucose control in gestational diabetes [23], exclusive breastfeeding [6,24], mental health [25-28], and maternal and perinatal mortality in rural and resource-poor settings [29-31]. The main features of MCH apps include health education, pregnancy planning, engagement with care, self-monitoring, and peer support. If women engage fully with these activities throughout pregnancy, it is plausible that this could improve important perinatal outcomes. However, evidence for this is limited [32,33].

China has a fast-growing market for MCH-related apps. A 2018 survey on maternal and infant apps identified 17 apps with more than 1 million monthly active users [34]. MCH apps in China are most frequently used for sharing parenting experiences, gestation-specific knowledge and tools, baby growth records, questions and answers about pregnancy and child health, social connections, and e-commerce [35]. In an in-depth review of market MCH apps conducted in 2018, we identified more than 6000 MCH-related apps in the Android and iOS app stores. Most are commercial apps, offering multiple features; however, despite their popularity, there is no published evidence regarding their effects on pregnancy outcomes [7].

### Objective

We hypothesized that app use during pregnancy would improve women’s engagement with formal maternity services and self-care and that this in turn would improve perinatal outcomes. The major objective of this study is to explore the use of MCH apps among pregnant Chinese women and explore the associations between app use and adverse outcomes at birth.

## Methods

### Study Design

This was a retrospective study investigating the associations between self-reported use of MCH apps and adverse pregnancy outcomes.

### Settings and Recruitment of Participants

Postnatal wards at 6 MCH hospitals were selected from Beijing and Hebei Province. The hospitals included 1 urban city hospital (Shijiazhuang), 4 suburban district hospitals (Huairou, Pinggu, Luquan, and Daxing), and 1 rural county hospital (Gaoyang). All women admitted to the selected postnatal ward at each study hospital were approached by a trained research physician before discharge. Women were eligible if they had delivered a singleton baby at >28 weeks’ gestation during the study period (from October 2017 to January 2018) and gave consent to participate in the study. Women with severe pre-existing disease such as cardiac disease, systemic lupus erythematosus, or malignant tumors were excluded because these conditions are independently associated with poorer perinatal outcomes.

### Data Collection for Events and Exposures

A structured questionnaire was designed to ascertain use of MCH apps during pregnancy (Multimedia Appendix 1). After piloting and refinement, the final questionnaire comprised 10 items (Multimedia Appendix 1), and it took 5-8 minutes to complete. The questionnaire was administered by trained research doctors in the postnatal wards to all the eligible women after the delivery of their baby, when, being the postpartum period, they were relatively relaxed and cooperative. Considering the good compliance, participation was voluntary, with no monetary or other incentives. Antenatal and perinatal outcomes were extracted directly from medical records into a case report form by the research doctors. Data covered in the case report form included (1) medical and pregnancy history, including previous pregnancy complications and adverse outcomes; (2) maternal antenatal screening results, including height, weight, and blood pressure; and (3) pregnancy outcomes, including gestational age at birth in weeks and days, delivery mode, birth weight in grams, and pregnancy and birth complications for the women and the babies.

Several measures were taken to avoid selection bias for both app use and pregnancy outcomes. We sequentially recruited all women from 1 general postnatal ward at each study hospital to minimize selection bias. Standard training was conducted for the research doctors at each hospital regarding study protocol and quality control for data collection. The participating women and research doctors were not aware of the study hypothesis. Regular site visits and data inspection were conducted by 2 inspectors from the research team to ensure the completeness and accuracy of outcome information in comparison with hospital records.

### Definition of MCH Apps, App Features, and User Categories

We defined an MCH app as any mobile phone app aiming to improve knowledge, behaviors, self-care, and antenatal health services use during pregnancy. Specific features of these apps include health education and promotion, physical or biodata monitoring, reminders, web-based counseling, communication, appointment making, laboratory result checking, and payments [3]. The app could be designed exclusively for use in pregnant women (MCH specialized app), or it could be a multipurpose app embedded with MCH features. We included both commercial and government and noncommercial apps.

The adopted features were counted for each app based on a previously published classification system of 23 categories [7]. The most relevant are health education, counseling, financial transactions and incentives, health status self-monitoring, reminders, appointment making, client-to-client communication, laboratory result checks, diary, shopping, games, and hospital service promotion. As most apps contained >1 feature, for the purpose of analysis, all apps were considered in aggregate.

**Figure 1:**
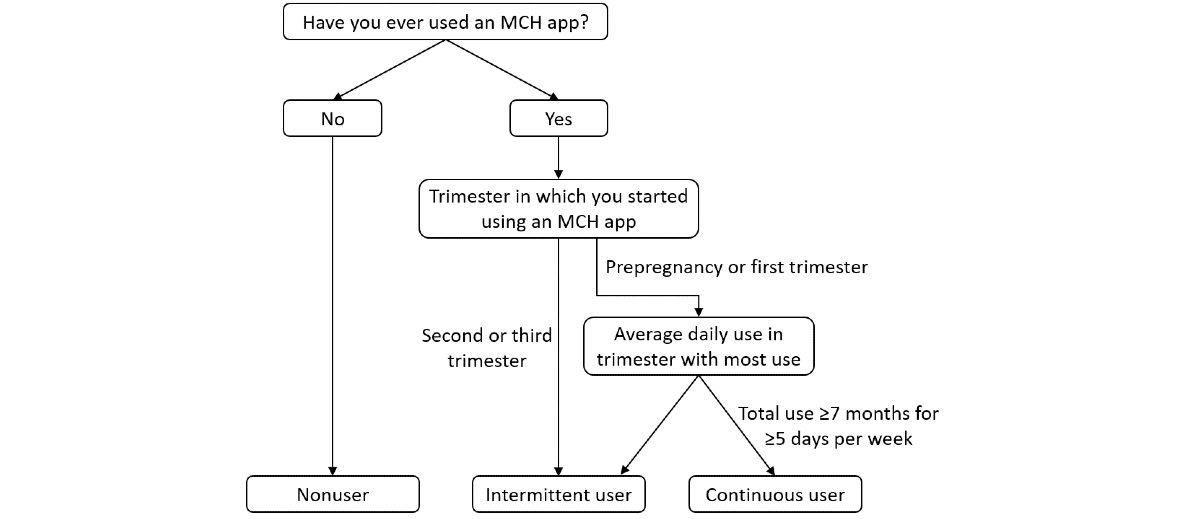
The categorization of women users of maternal and child health apps. MCH: maternal and child health.

Figure 1 shows the procedure we followed to categorize the women according to their responses to the following questions: Have you ever used an MCH app during pregnancy? When (which trimester) did you start using it? How long have you been accumulatively using the app since starting use (month)? Which trimester did you use the MCH app most? How many days did you use it every week during the most frequently used trimester? In summary, nonusers were defined as women who did not use any MCH app. App users were further categorized into continuous users if they reported using ≥1 MCH apps almost every day during the entire pregnancy and intermittent users if they reported less frequent or shorter durations of use.

### Definitions of Outcomes

Because of the anticipated low incidence of severe adverse pregnancy outcomes, a composite adverse pregnancy outcome (CAPO) was used as the primary outcome for minimum sample size estimation and analysis. The CAPO comprised premature birth (delivery at ≤37 completed weeks of gestation), low birth weight (<2500 g), birth defects (Chinese National Criteria of Birth Defects and Tiny Deformities [36]), stillbirth (World Health Organization definition [37]), and neonatal asphyxia (experts’ consensus in China [38]). The CAPO was developed as an unweighted index, with a case being defined as ≥1 of any of the aforementioned adverse outcomes assessed. The diagnosis of the individual pregnancy outcome was made by obstetricians at the participating hospitals during routine practice.

The exploratory outcomes included (1) CAPO incidence among 3 user groups (nonusers, intermittent users, and continuous users), (2) incidence of macrosomia (baby weighing ≥4000 g), and (3) incidence of each of the component outcomes of CAPO.

### Sample Size and Statistical Analysis

We estimated the CAPO rate in this population to be 15% to calculate the sample size. This was based on the reported incidences of the component outcomes of CAPO, including low birth weight (8.1%) [39], preterm birth (6.94%) [40], neonatal asphyxia (3.78%) [41], birth defects (1.92%) [36], and stillbirth (0.95%) [42], in China. Assuming that the estimated baseline CAPO rate is 15% and most participants (we assumed two-thirds) are MCH app users, at least 1800 participants (600 nonusers and 1200 overall users) would need to be recruited to explore an absolute decrease in CAPO by 5% for MCH app users compared with nonusers, with a power of no less than 80% and significance level of 5%, calculated by PASS 11 software (NCSS, LLC).

The characteristics of the participants were described as categorical variables and expressed as numbers and percentages. The proportion of women who started using an MCH app and used an app most frequently within each pregnancy trimester was described as a bar graph. The Pearson chi-square test was performed to compare the difference in incidence of CAPO (primary outcome) and other indicators (exploratory outcomes) between MCH app users and nonusers and among the 3 app use groups. Logistic regression analysis was performed to analyze associations between the use of MCH apps and pregnancy outcomes after controlling for confounders, including hospital, age, education level, household income level, parity, gravidity, and history of cesarean section. Abortion was excluded in the model because of its strong collinearity with gravidity and parity. History of hypertension and diabetes was also excluded to avoid overadjustment because blood pressure and blood glucose level might be on the causal chain from app use to CAPO. We did not use log binomial regression and Poisson regression because of convergence issues and because the log-likelihood value cannot be further improved, respectively. Heterogeneity among the different hospitals (the 6 hospitals) and 2 hospital levels (Shijiazhang city hospital and other suburban and rural hospitals) was analyzed by examining the interaction effect of (hospital)×(app user group) and (hospital level)×(app user group), respectively, based on the logistic regression model. We conducted a complete case analysis, with no imputation for missing variables. Sensitivity analyses were conducted to determine if there was any association of app brand, gestational age when app use was started, and intensity of use (continuous daily use vs intermittent use) with CAPO and macrosomia. All data analyses were performed with SAS (version 9.4; SAS Institute).

### Ethical Approval and Informed Consent

The study protocol was approved by the ethics committee of Peking University on September 19, 2017. Verbal informed consent was obtained from all participants included in the study. Identifiable personal information was not collected; all data were kept confidential and safe according to the internal data security policy of the George Institute for Global Health, and they were only accessible to authorized researchers.

## Results

### Characteristics of Participants

During the recruitment period, there were 1882 postnatal women admitted to the participating wards at the 6 hospitals. Of the 1882 women, we excluded 7 (0.4%) who refused participation, 10 (0.5%) who had multiple pregnancies, 13 (0.7%) who delivered at <28 weeks’ gestation, and 2 (0.1%) who had severe disease, leaving 1850 women in the study. The participants were aged 18-45 years, with an average age of 29.6 (SD 4.4) years. Detailed characteristics of the participants are described in the nonuser and user (continuous and intermittent) categories in Table 1.

Approximately three-fourths (1393/1850, 75.3%) of the women reported using MCH apps during pregnancy. Of the 1393 users, 876 (62.9%) were intermittent users and 517 (37.1%) were continuous users. Apart from the women at Pinggu MCH hospital, where an app was required for antenatal appointments, most of the other participants fell into the category of intermittent users. Age, education, household monthly income, gravidity, parity, history of cesarean section, and incidence of gestation diabetes differed (*P*<.001) among the MCH app user groups. In general, nonusers had less education and household income, were older, and had experienced more previous pregnancies (Table 1).

**Table 1:** Characteristics of participants by maternal and child health (MCH) app user groups (N=1850).

Characteristics		Total, n (%)	Nonusers (n=457), n (%)	Intermittent users (n=876), n (%)	Continuous users (n=517), n (%)
**MCH hospitals^a^**					
	Huairou	254 (13.7)	61 (24)	141 (55.5)	52 (20.5)
	Gaoyang	267 (14.4)	105 (39.3)	104 (39)	58 (21.7)
	Pinggu	347 (18.8)	92 (26.5)	46 (13.3)	209 (60.2)
	Luquan	243 (13.1)	32 (13.2)	188 (77.4)	23 (9.5)
	Daxing	257 (13.9)	35 (13.6)	162 (63)	60 (23.3)
	Shijiazhuang	482 (26.1)	132 (27.4)	235 (48.8)	115 (23.9)
**Age^a^ (years)**					
	18-24	228 (12.3)	36 (15.8)	114 (50)	78 (34.2)
	25-34	1351 (73)	321 (23.8)	648 (48)	382 (28.3)
	35-45	271 (14.6)	100 (36.9)	114 (42.1)	57 (21)
**Education level^a^**					
	Middle school or below	475 (25.7)	199 (41.9)	178 (37.5)	98 (20.6)
	High school	451 (24.4)	101 (22.4)	235 (52.1)	115 (25.5)
	College	487 (26.3)	81 (16.6)	259 (53.2)	147 (30.2)
	University or above	437 (23.6)	76 (17.4)	204 (46.7)	157 (35.9)
**Household monthly income^a^**					
	<RMB ¥3000^b^	331 (17.9)	119 (36)	159 (48)	53 (16)
	RMB ¥3000-RMB ¥4999^c^	872 (47.1)	216 (24.8)	415 (47.6)	241 (27.6)
	RMB ¥5000-RMB ¥9999^d^	475 (25.7)	89 (18.7)	232 (48.8)	154 (32.4)
	≥RMB ¥10,000^e^	172 (9.3)	33 (19.2)	70 (40.7)	69 (40.1)
**Gravidity^a^**					
	1	493 (26.6)	64 (13)	279 (56.6)	150 (30.4)
	2	586 (31.7)	139 (23.7)	272 (46.4)	175 (29.9)
	3	453 (24.5)	137 (30.2)	194 (42.8)	122 (26.9)
	≥4	318 (17.2)	117 (36.8)	131 (41.2)	70 (22)
**Parity^a^**					
	0	695 (37.6)	84 (12.1)	364 (52.4)	247 (35.5)
	1	1076 (58.2)	333 (30.9)	481 (44.7)	262 (24.3)
	≥2	79 (4.3)	40 (50.6)	31 (39.2)	8 (10.1)
**History of cesarean section^a^**					
	No	1431 (77.4)	320 (22.4)	686 (47.9)	425 (29.7)
	Yes	419 (22.6)	137 (32.7)	190 (45.3)	92 (22)

^a^*P*<.001 for differences among the subgroups based on Pearson chi-square test.

^b^US $472.2.

^c^US $472.2-US $786.8.

^d^US $787-US $1573.8.

^e^US $1574.

### MCH App Use

Among the 1393 MCH app users, 1003 (72%) used 1 app, 319 (22.9%) used 2 apps, and 71 (5.1%) used 3 apps during pregnancy. In total, 127 MCH apps were reported as being used. The most popular app was *Baby Tree*, which was used by 51.3% (715/1393) of the women; followed by *Meet You*, 27.6% (385/1393); *Pregnant Partner*, 10.3% (144/1393); *Daxing MCH Hospital WeChat Official Account*, 10.1% (140/1393); *Mom Bang*, 5% (69/1393); and others, 28.8% (401/1393). *Daxing MCH Hospital WeChat Official Account* is a not-for-profit applet developed by the MCH hospital and based on a social media platform, *WeChat*, and exclusively used by pregnant women registered at Daxing Hospital. The rest were commercial apps.

All the top 5 apps included health education and promotion as well as payment features. Except for Daxing MCH Hospital WeChat Official Account, the apps also included features related to antenatal reminders, health status self-monitoring, peer communication, counseling, and diary. Most (1136/1393, 81.6%) of the women app users used ≥2 features. The top 3 app features used were health education (1393/1393, 100%), health status self-monitoring (755/1393, 54.2%), and antenatal clinic appointment reminders (602/1393, 43.2%). Multimedia Appendix 2 shows the detailed features of the 5 most frequently used apps.

Among the MCH app users, most women started using the apps quite early during the pregnancy, with approximately 1 in 5 (267/1393, 19.2%) starting before pregnancy, two-thirds (921/1393, 66.1%) starting during the first trimester, and only a small proportion (205/1393, 14.7%) starting later during the second and third trimesters. As their pregnancy progressed, women reported more frequent use of the apps. The proportion of women who reported using the app most frequently increased from 4.2% (58/1393) during prepregnancy to 24.8% (345/1393), 37.3% (519/1393), and 44.7% (623/1393) during the first, second, and third trimester, respectively.

### Association Between MCH App Use and Pregnancy Outcomes

There were 119 babies with a CAPO rate of 6.4% among the 1850 participants. Although continuous users had the lowest incidence of CAPO (26/517, 5%), no statistical difference was found for incidence of CAPO (primary outcome) between all users (88/1393, 6.3%) and nonusers (31/457, 6.8%; *P*=.73) or between nonusers (31/457, 6.8%) and intermittent users (62/876, 7.1%) or continuous users (26/517, 5%; *P*=.31). Of the 9.2% (169/1850) of the babies born macrosomic, the incidence of macrosomia was similar among intermittent users (83/876, 9.5%) and continuous users (49/510, 9.6%), which, although higher than that in nonusers (37/457, 8.1%), did not reach statistical significance (*P*=.37). The incidence of low birth weight was the lowest in the continuous users among the 3 groups (*P*=.04), but no statistical significance was found among the groups for the other component outcomes of CAPO (Table 2).

Table 3 shows the odds ratios (ORs) of CAPO and macrosomia among different MCH app users based on logistic regression analysis with adjustment for hospital, age, education level, household income level, parity, gravidity, and history of cesarean section. No significant difference was found for CAPO incidence when comparing continuous users and intermittent users and nonusers (OR 0.77, 95% CI 0.48-1.25) or comparing continuous users and nonusers (OR 0.77, 95% CI 0.42-1.42). A positive association was detected between MCH app use and the odds of delivering a macrosomic baby (OR for any app use compared with none 1.44, 95% CI 0.95-2.17, and OR for continuous app use compared with none 1.55, 95% CI 0.91-2.63); however, a null effect cannot be excluded.

The heterogeneity analyses did not show any significant findings for either CAPO or macrosomia. The *P* values for the interaction effect of 6 hospitals×2 app user groups (user vs nonuser) were .75 for CAPO and .49 for macrosomia, whereas the *P* values for the interaction effect of 2 hospital levels×2 app user groups (user vs nonuser) were .32 for CAPO and .31 for macrosomia.

Finally, we conducted sensitivity analyses to explore associations of app brand name (Multimedia Appendix 2), starting time, and use frequency with pregnancy outcomes, including macrosomia. No statistically significant association was detected between app use and pregnancy outcomes, although the risk of macrosomia seemed to be increased among users who used certain apps (*Mei You* vs other apps: OR 1.53, 95% CI 0.92-2.54) and among those who began using the apps at an earlier stage (prepregnancy vs second and third trimester: OR 1.34, 95% CI 0.66-2.74; first trimester vs second and third trimester: OR 1.41, 95% CI 0.80-2.51; Multimedia Appendix 3).

**Table 2:** Adverse pregnancy outcomes among different app user groups (N=1850).

Adverse pregnancy outcomes	Total (N=1850), n (%)	Nonusers (n=457; user group 1), n (%)	Users				*P* value^a^ (comparison between user groups 1 and 2)		*P* value^b^ (comparison among user groups 1, 3, and 4)
			All users (n=1393; user group 2), n (%)	Intermittent users (n=876; user group 3), n (%)	Continuous users (n=517; user group 4), n (%)				
CAPO^c^	119 (6.4)	31 (6.8)	88 (6.3)	62 (7.1)	26 (5)	.73		.31	
Premature birth	75 (4.1)	21 (4.6)	54 (3.9)	39 (4.5)	15 (2.9)	.50		.29	
Low birth weight	34 (1.8)	7 (1.5)	27 (1.9)	23 (2.6)	4 (0.8)	.57		.04	
Birth defects	22 (1.2)	3 (0.7)	19 (1.4)	12 (1.4)	7 (1.4)	.23		.48	
Stillbirth	3 (0.2)	1 (0.2)	2 (0.1)	0 (0)	2 (0.4)	.73		.21	
Neonatal asphyxia	9 (0.5)	4 (0.9)	5 (0.4)	4 (0.5)	1 (0.2)	.17		.31	
Macrosomia^d^	169 (9.2)	37 (8.1)	132 (9.5)	83 (9.5)	49 (9.6)	.37		.67	

^a^On the basis of the Pearson chi-square test.

^b^On the basis of the Pearson chi-square test. No pairwise Pearson comparison was conducted because no significant difference was found for overall comparison for each outcome.

^c^CAPO: composite adverse pregnancy outcome, defined as a case with ≥1 event of premature birth, low birth weight, birth defects, stillbirth, and neonatal asphyxia.

^d^Not a component of composite adverse pregnancy outcome.

**Table 3:** Odds ratios (ORs) of CAPO (composite adverse pregnancy outcome) and macrosomia among different maternal and child health app users: results of logistic regression analysis^a^.

Comparison	CAPO^b^		Macrosomia	
	OR (95% CI)	*P* value	OR (95% CI)	*P* value
User vs nonuser	1.04 (0.66-1.64)	.87	1.44 (0.95-2.17)	.09
Continuous user vs intermittent user and nonuser	0.77 (0.48-1.25)	.29	1.22 (0.82-1.82)	.32
Continuous user vs nonuser^c^	0.77 (0.42-1.42)	.40	1.55 (0.91-2.63)	.11

^a^Controlling for hospital, age, education, household income, parity, gravidity, and history of cesarean section.

^b^Defined as any pregnancy outcome of premature birth, low birth weight, birth defects, stillbirth, and neonatal asphyxia.

^c^A total of 876 intermittent users were excluded.

## Discussion

### Principal Findings

In this retrospective multicenter study in north China, we found that MCH app use is common and many women start using apps in early pregnancy. In this study, the top 3 features of apps reported were health education (1393/1393, 100%), health status self-monitoring (755/1393, 54.2%), and antenatal clinic appointment reminders (602/1393, 43.2%). However, it is notable that app use was not common to all women, with nonusers having less education, lower income, and higher age and parity compared with users (*P*<.01). We did not observe any association between app use and the risk of CAPO.

Although some studies have shown that MCH app use can provide benefits, including reducing health anxiety, improving satisfaction with pregnancy care, and helping women make better lifestyle and nutritional choices [43-45], few studies have evaluated the effects of market MCH apps on pregnancy outcomes. In this study, we did not observe any association between app use and the risk of CAPO. This could be due to several reasons. First, the download and reported use of an MCH app does not necessarily result in behavior changes. Second, as all women attending these hospitals also had access to free prenatal education sessions that target self-care and health education, any additional benefit from app use was likely to have been marginal. Third, the observational design had a weakness in balancing potential confounding factors between app users and nonusers and in controlling selection and recording bias. Fourth, heterogeneity in the association may exist among different MCH apps, different app users, different regions, and different hospital levels, which might dilute the effects of the outcome. Finally, the sample size was not powerful enough to detect the benefit of app use, although our study showed that incidences of CAPO, premature birth, low birth weight, and neonatal asphyxia were the lowest among continuous users (Table 3). A large study, especially a well-designed trial, could be a solution in terms of clarifying the effect of app use and potential heterogeneities.

Reassuringly, we did not find any evidence of harms. It is notable that none of the top 5 apps reported by the women in this study were disease- or condition-specific. It is likely that apps targeting specific behaviors where there is evidence of benefit regarding outcomes from nonapp studies could be more effective than generic apps for pregnant women. Some good examples are apps used to improve blood glucose management in women with gestational diabetes [23] and apps tracking the menstrual cycle to improve fecundability [46]. Ideally, apps should be developed using theories of behavior change, accompanied by evaluation before recommendation for general use [8].

### Use of MCH Apps

MCH apps are gaining popularity worldwide. The number of downloads for such apps has reached hundreds of millions [6-9]. However, few studies report MCH app use among pregnant women in the real world with considerations of multiple app use. In this study, we found that approximately three-fourths (1393/1850, 75.3%) of the pregnant women used ≥1 MCH apps in north China and more than one-fourth (387/1393, 27.8%) used an app every day. This popularity level is higher than that reported in other countries that promote app use, such as Jordan and South African [47,48]. Considering that more than 99% of the pregnant women in China deliver at county- or higher-level hospitals [49] and these were the target hospitals for recruitment in this study, our results on app coverage should have good representativeness for pregnant women in northern China. The high use rates we report indicate that pregnant women have a very high acceptance of MCH apps, in keeping with a market analysis conducted in China by Forward-The Economist, where adherence to MCH apps was reported as rising, with 44.1% users logging in every day in 2019 [50]. A cross-sectional study found that commercial MCH apps with more favorable user experience, in-app purchases, and in-app advertisements were more frequently downloaded [51]. However, although we observed a wide range of app functions, we are not able to comment on whether any of these helped the women in our study to achieve more downloads and sustain behavior changes.

Disparities or inequities regarding MCH outcomes between rural and urban areas and across geographical regions have been noticed and narrowed significantly by the Chinese government [52]. However, attention should be paid to emerging challenges arising from mHealth access inequities. Although mHealth technology in MCH care has spread quickly, social disparities in access and frequency of use exist and need to be dealt with. Although an association between MCH app use and improved pregnancy outcomes has not been shown, women with lower education and household income and those with more children were less likely to use apps in this study. If apps are to be used in pregnancy care pathways, programs need to ensure equitable and universal coverage. In addition, although our study did not confirm the existence of heterogeneity among hospitals and hospital levels, further exploration is still worthwhile in future studies.

### Most Common Features of MCH Apps

In this study, the mostly commonly used features of MCH apps were health education, health status self-monitoring, antenatal appointment reminders, communication, appointment making, laboratory result checking, shopping, counseling, diary, and financial transactions. The most popular apps such as *Baby Tree* and *Meet You* offered most of these features, with the exception of the facility to make antenatal appointments or check laboratory results. These findings were similar to those we found in our market review of MCH apps and the maternal and infant industry report in 2020 [7,50]. Notably, no apps were described with decision support functions, for example, for management of gestational diabetes, healthy weight gain, exercise in pregnancy, blood pressure, and preeclampsia [53].

### App and Outcome Selection

We chose to focus on all MCH apps in aggregate, rather than specific apps, because we wanted to explore whether there were any general effects on pregnancy outcomes. There were 2 major reasons for adopting this approach. First, many pregnant women use multiple apps (in this study, 28%, 390/1393, of the women used ≥2 apps during pregnancy), making it hard to differentiate the effects of certain apps in an observational study. Second, most MCH apps have multiple and similar feature components, as shown in Multimedia Appendix 2. For the purposes of this study, we therefore assumed that the effects of the apps would be similar.

Although we observed no statistical difference in CAPO between MCH app users and nonusers or among the 3 user groups, we observed a (nonsignificant) trend toward increased macrosomia with app use. Whether this can be attributed to effects secondary to behavior changes resulting from use of the apps or to these women making improved nutritional choices or whether this reflects the better social standing of the women who used the apps merits further exploration.

### Limitations

As this was a retrospective study, we were unable to control for reporting or recall bias among certain groups. As the women and the data collectors were unaware of the study hypothesis, we have assumed that any such bias would have been evenly spread among the MCH app user groups.

The observed incidence of CAPO (6.4%) at the study hospitals was much lower than what we estimated (15%), which meant that this study was underpowered to detect the difference in CAPO. In contrast, the incidence of macrosomia (9.2%) in this study was higher than previously reported in 2018 in China (2.5%) [39]. Further studies could limit the exposure to studying only the most popular apps, with prospective or randomized designs.

We have presented several exploratory analyses, including comparison of different outcome indicators among different user groups, with the purpose of guiding future studies in this area. This may have increased the chance of significant findings (type I error); we did not conduct any adjustment to control the error.

Finally, although nearly all the apps cover features regarding MCH care and we used MCH app as the general name for such apps, the evaluation was only conducted among women soon after the delivery of their baby and while they were still at the hospital. As a result, the use of parenting features and the effect on children’s health were not evaluated in this study.

### Conclusions

MCH apps are widely used among pregnant women in China, but the general effects of such apps on severe perinatal outcomes have not been well evaluated worldwide. Our retrospective study showed no significant benefit of MCH apps in improving overall adverse pregnancy outcomes. The findings on coverage of MCH apps, app adherence, and observed effect in reducing or increasing adverse pregnancy outcomes are informative for future study design and app development and upgrade.

We believe that the findings of this study have important implications for researchers, clinicians, and end users. For researchers, the wide reach of apps among pregnant women could be a powerful tool for public health and health promotion; however, the lack of effect that we have demonstrated points to a need for further research to understand the mechanisms of action of these apps (ie, behavior change) and determine how apps can be used as a tool to strengthen delivery of maternity care. For clinicians, this paper demonstrates the high rate of app uptake among pregnant women and thus the importance of asking women about what apps they are using and signposting them to apps that are evidence-based. For end users, a signposting system to rank the clinical accuracy of apps could be valuable, although how this would work in practice in a very crowded marketplace needs to be assessed with further work.

## Appendix

Multimedia Appendix 1 Questionnaire for the use of a maternal and child health app among pregnant women.

Multimedia Appendix 2 Major features adopted by top 5 apps.

Multimedia Appendix 3 Associations of composite adverse pregnancy outcome and macrosomia with utility of maternal and child health apps among 1393 app users: results of logistic regression analysis.

## Abbreviations

CAPO: composite adverse pregnancy outcome
MCH: maternal and child health
mHealth: mobile health
OR: odds ratio
