# Supplementary material for: Mobile Phone App Use Among Pregnant Women in China and Associations Between App Use and Perinatal Outcomes: Retrospective Study
Source: JMIR Form Res. 2022 Jan 25;6(1):e29644. doi: 10.2196/29644 (PMC8826146; doi:10.2196/29644)
Supplement: Multimedia Appendix 1 [file formative_v6i1e29644_app1.docx]

## Appendix 1: Questionnaire for the use of a maternal and child health app among pregnant women

**Hospital No.____** **Patient No**

**Note;** the questions items are single choice ones when not marked as "multiple choice"; tick the selected option(s).

**A. basic information**

1. Education: ①primary school and below ②middle school ③high school ④college ⑤university and above
2. Monthly family income（yuan）: ①＜3,000 ②3,000-5,000 ③5,000-10,000 ④10,000-20,000 ⑤＞20,000

**B. usage of MACH app(s) during this pregnancy**

1. Have you ever used mobile phone apps for pregnancy preparation, pregnancy or parenting（hereinafter referred as MCH App）？

① yes，skip to question 5 ②no

| Reason for not using MCH App: （multiple choice）  ①too troublesome ② I have known the knowledge  ③there is no service I need on MCH App ④ I don’t know MCH App  Skip to question 11 |
| --- |

1. Names of your most frequently used app:

*note: if it is Wechat public account, please provide the name of Wechat public account and mark“**wx”** after the name.

①_______________ ②_________________ ③__________________

1. When did you start the usage of MCH App？

①before pregnant ②first trimester (before gestation week 12)

③second trimester (gestational 12-28) ④third trimester (gestational 28-41)

1. Accumulated time of use in total: _______month(s)

*note : Accumulated time refers the total time from the first day of your use of the App to the day of the interview; if there was a period of time(more than 3 months) you did not use the App, the period of time should be deducted from the total time of use.

1. Which trimester did you use MCH App most frequently？

①before pregnant ②first trimester (before gestation week 12)

③second trimester (gestational 12-28) ④third trimester (gestational 28-41)

1. How many days did you use every week during the most frequently used trimester？

① <= 1 day/week ②2-3 day/week ③4-5 day/week ④almost everyday

1. Which feature did you use most often？(multiple choice)

Health Education: ①pregnancy preparation ②pregnancy ③parenting ④none

Tools: ①Menstrual cycle and ovulation records ②ANC reminder ③FHR/fetal movement monitor ④weight management (incl. weight record and diet instruction) ⑤Blood pressure management (incl. BP record, reminder of abnormal value, life style and medication instruction）⑥Blood glucose management (incl. BG record, reminder of abnormal value, and medication instruction ⑦others_____ ⑧none

Hospital service: ①appointment ②lab result check ③communication with doctors ④none

Social tools: ①community and chat ②dairy or recording ③payment ④shopping ⑤none

1. Will you use MCH App after your delivery？

① yes ② no

Thank you for your participation！

Interviewer name: _____________

Date: __dd/mm/year
