# Supplementary material for: Mobile Phone App Use Among Pregnant Women in China and Associations Between App Use and Perinatal Outcomes: Retrospective Study
Source: JMIR Form Res. 2022 Jan 25;6(1):e29644. doi: 10.2196/29644 (PMC8826146; doi:10.2196/29644)
Supplement: Multimedia Appendix 2 [file formative_v6i1e29644_app2.docx]

## Appendix 2: Major features adopted by top 5 apps

| Features ^a^ | Top 5 apps | | | | | User  proportion  n (%) ^c^ |
| --- | --- | --- | --- | --- | --- | --- |
|  | *Baby*  *tree* | *Meet*  *you* | *Daxing*  *MCH* ^b^ | *Pregnant*  *partner* | *Mom*  *bang* |  |
| No of users, n (%) | 905  (64.5) | 428  (30.5) | 204  (14.5) | 126  (9.0) | 82  (5.8) | 1393 |
| Health education | √ | √ | √ | √ | √ | 1393(100.0) |
| Pregnancy knowledge | √ | √ | √ | √ | √ | 1314(94.3) |
| Parenting knowledge | √ | √ | √ | √ | √ | 556(39.9) |
| Health status self-monitoring | √ | √ | x | √ | √ | 755(54.2) |
| Fetal heart rate | √ | √ | x | √ | √ | 320(23.0) |
| Weight management | √ | √ | x | √ | √ | 267(19.2) |
| Menstrual cycles &  ovulation records | √ | √ | x | √ | √ | 202(14.5) |
| Reminder | √ | √ | x | √ | √ | 602(43.2) |
| Client-to-client communication | √ | √ | x | √ | √ | 290(20.8) |
| Appointment making | x | x | √ | x | x | 213(15.3) |
| Laboratory results check | x | x | √ | x | x | 199(14.3) |
| Shopping | √ | √ | x | √ | √ | 141(10.1) |
| Counselling | √ | √ | x | √ | √ | 123(8.8) |
| Diary | √ | √ | x | √ | √ | 99(7.1) |
| Financial transaction and incentive | √ | √ | √ | √ | √ | 64(4.6) |
| Hospital service promotion | √ | x | √ | x | x | 0(0.0) |

a: The features were previously defined by a study for 23 categories, (6) and only adopted ones listed here.

b: Except for *Daxing MCH* developed as a hospital WeChat official account, all the other four apps were traditional mobile apps.

c: Each participant might use more than one feature; the sum proportion may exceed 100 percent.
