# Supplementary material for: Mobile Phone App Use Among Pregnant Women in China and Associations Between App Use and Perinatal Outcomes: Retrospective Study
Source: JMIR Form Res. 2022 Jan 25;6(1):e29644. doi: 10.2196/29644 (PMC8826146; doi:10.2196/29644)
Supplement: Multimedia Appendix 3 [file formative_v6i1e29644_app3.docx]

**Appendix 3 Associations of composite adverse pregnancy outcome and macrosomia with utility of maternal and child health apps among 1393 app users: results of logistic regression analysis ^a,b^**

| App utility | CAPO ^b^ | | |  | Macrosomia | | |
| --- | --- | --- | --- | --- | --- | --- | --- |
|  | OR | 95%CI | P |  | OR | 95%CI | P |
| Most frequently used apps (vs others) | | | | | | | |
| *Baby Tree* | 0.89 | (0.54-1.48) | 0.657 |  | 1.00 | (0.63-1.60) | 0.996 |
| *Meet You* | 0.65 | (0.33-1.27) | 0.204 |  | 1.53 | (0.92-2.54) | 0.105 |
| Starting time of app use (vs 2^nd^/3^rd^ trimester) | | | | | | | |
| Pre-pregnancy | 0.722 | (0.31-1.67) | 0.448 |  | 1.34 | (0.66-2.74) | 0.418 |
| 1^st^ trimester | 1.07 | (0.55-2.10) | 0.839 |  | 1.41 | (0.80-2.51) | 0.239 |
| Frequency of use (vs intermittent use) | | | | | | | |
| Continuous use | 0.72 | (0.42-1.24) | 0.235 |  | 1.11 | (0.71-1.75) | 0.648 |

*a: Controlling for hospital, age, education, household income, parity, gravidity and history of C-section.*

*b: CAPO was defined as any pregnancy outcome of premature birth, low birth weight, birth defects, stillbirth and neonatal asphyxia.*
